# Interferon Regulatory Factor 9 Structure and Regulation

**DOI:** 10.3389/fimmu.2018.01831

**Published:** 2018-08-10

**Authors:** Alvin Paul, Thean Hock Tang, Siew Kit Ng

**Affiliations:** Advanced Medical and Dental Institute, Universiti Sains Malaysia, Penang, Malaysia

**Keywords:** interferon regulatory factor 9, JAK-STAT, type I interferons, innate immunity, interferon-stimulated genes, antiviral defense

## Abstract

Interferon regulatory factor 9 (IRF9) is an integral transcription factor in mediating the type I interferon antiviral response, as part of the interferon-stimulated gene factor 3. However, the role of IRF9 in many important non-communicable diseases has just begun to emerge. The duality of IRF9’s role in conferring protection but at the same time exacerbates diseases is certainly puzzling. The regulation of IRF9 during these conditions is not well understood. The high homology of IRF9 DNA-binding domain to other IRFs, as well as the recently resolved IRF9 IRF-associated domain structure can provide the necessary insights for progressive inroads on understanding the regulatory mechanism of IRF9. This review sought to outline the structural basis of IRF9 that guides its regulation and interaction in antiviral immunity and other diseases.

## Introduction

Interferon regulatory factor 9 (IRF9) was first discovered as part of a protein subunit purified from the interferon-stimulated gene factor 3 (ISGF3) complex (1). Early studies have referred IRF9 as ISGF3γ and p48—due to its molecular weight of 48 kDa (1–4). IRF9 is best characterized as a transcription factor that mediates (as part of ISGF3) the type I interferon (IFN) response by regulating the downstream expression of interferon-stimulated genes (ISGs) (5, 6). IRF9 is also involved in regulating cell proliferation (4), tumor formation (7), cardiovascular disease (8), inflammation (9), autoimmune disease (10), and immune cell regulation (11), some of which is not related to ISGF3 complex.

There are nine known members of IRF family in humans; numerically designated IRF1 to IRF9 [reviewed in Ref. (12–15)]. Major functions of IRFs involve transcriptional regulation of the immune system and cell growth. All IRFs share three common domains; an N-terminal helix-turn-helix DNA-binding domain (DBD) containing five conserved tryptophan repeats; a C-terminal IRF-associated domain (IAD) responsible for protein–protein interactions [(5), reviewed in Ref. (14, 16)]; and a linker region. It has been suggested that the ancestral gene of IRFs was already present in the last common ancestor of Metazoa, thus tying the evolution of IRF family with that of multicellular animals (17). The IRF family then further diverge evolutionarily along with the adaptive immune system that emerged in early vertebrates, as reflected in their role at the innate-adaptive immunity interface (18).

IRF1 and IRF2 were the first IRFs to be identified where early studies indicated a “*yin-yang*” relationship of the two, functioning as activator and repressor of IFNα/β genes, respectively (19). IRF3 and IRF7 are important regulators in the type I IFN signaling. IRF3 functions to induce IFN-β genes during the first phase of type I IFN activation and binds with IRF7 in the second phase to induce IFN-α (20). A seminal study by Honda et al. (21) showed that homozygous deletion of *irf7* in mice exhibited no expression of type I IFN genes following viral infection, which indicates a definitive role of IRF7 in IFN signaling. Similarly, IRF5 is also involved in the induction of IFN response. IRF5 is activated downstream through the toll-like receptor (TLR)-MyD88 signaling and TRIF pathway to activate proinflammatory cytokine genes (22, 23). IRF4—expressed primarily in lymphoid cells—is known to interact with the PU.1 transcription factor to regulate the development of hematopoietic cells (24). Similarly, IRF8 is primarily expressed in hematopoietic cells and interacts with PU.1 to regulate IL-18 gene expression (25). Meanwhile IRF6 is required in the regulation of keratinocyte development (26) but its function in innate immunity is not known. Although the role of IRF6 in immune response is undefined, *IRF6* gene mutation in humans could lead to genetic disorders such as Van der Woude syndrome (27) and popliteal pterygium syndrome (28).

Interferon regulatory factor 9 was once dubbed “The forgotten IRF” by Paun and Pitha due to relative lack of studies compared to other IRFs (13). Though, recent advances point toward its apparent conflicting roles in health and diseases [reviewed in Ref. (29)]. A focused review by Suprunenko and Hofer (30) provided an excellent view on the overarching role of IRF9 in biological processes. Here, we attempt to explain on how the structural basis of IRF9 influence its regulation and function. We also briefly discuss the latest relevant research toward understanding of IRF9 beyond its role in ISGF3. This is imperative as IRF9 is increasingly implicated in other conditions beyond Janus kinase–signal transducer and activator of transcription (JAK–STAT) signaling (31).

## IRF9 Signaling in JAK–STAT Pathway

Activation of the type I IFNs response is mediated *via* JAK–STAT pathway, in a biphasic manner, as described in a compelling perspective review [reviewed in Ref. (32)]. The innate immune recognition of cells can occur in an intrinsic or extrinsic manner *via* pattern recognition receptors (PRRs) [reviewed in Ref. (33)]. Intrinsic recognition occurs in infected cells through PRRs such as NOD-like receptors and RIG-I-like receptors [reviewed in Ref. (34)]. Meanwhile, extrinsic recognition occurs in non-infected immune cells (e.g., macrophages and plasmacytoid dendritic cells) *via* PRRs such as the Toll-like receptors and C-type lectins [reviewed in Ref. (34)]. Both can lead to the induction of many cytokines, including type I IFNs (i.e., IFN-α and IFN-β). In the initial activation phase of innate antiviral immune response, activated TLR induces the production of early phase NF-κB-dependent proinflammatory cytokines, the mitogen-activated protein kinases, and the IRF-dependent antiviral cytokines (i.e., type I IFNs) [reviewed in Ref. (35)]. In the following phase, the secreted type I IFN induces an increased expression of ISGs in surrounding cells *via* JAK–STAT pathway.

**Figure 1:**
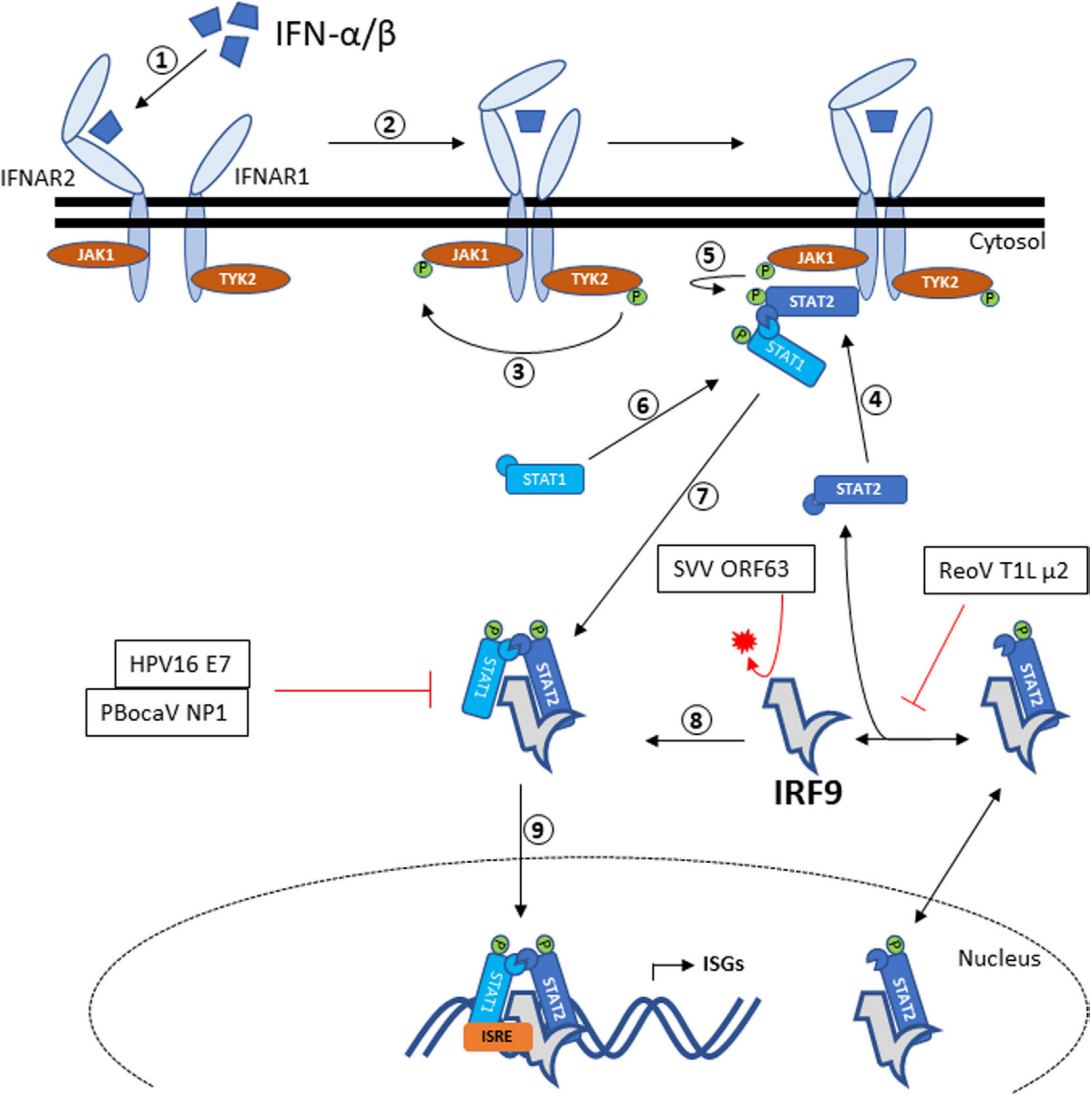
IRF9 signaling *via* the JAK–STAT pathway and antagonism by viral proteins. Recognition of IFN-α/β by IFNAR2 will trigger heterodimerization to IFNAR1, resulting in autophosphorylation of TYK2. Activated TYK2 then phosphorylates the adjacent JAK1. STAT2 recruited by activated IFNAR2 will be phosphorylated by JAK1, thus allowing docking of STAT1 that in turn gets phosphorylated. The phosphorylated STAT1–STAT2 heterodimer then dissociates from the IFNARs and forms the ISGF3 complex with IRF9. ISGF3 complex is then translocated into the nucleus and bind to the ISRE promoter sequence to initiate the transcription of ISGs. IRF9 has been shown to associate with STAT2 and shuttles between the cytoplasm and nucleus. Also annotated are the viral antagonisms directed toward IRF9. HPV16 E7, ReoV T1L μ2, and PBocaV NP1 binds to IRF9 and therefore prevents the formation of ISGF3 complex. Meanwhile, SVV ORF63 directs the proteasomal degradation of IRF9. Abbreviations: HPV16 E7, human papillomavirus 16 E7; SVV ORF63, simian varicella virus ORF63; ReoV T1L μ2, reovirus T1L μ2; PBocaV NP1, porcine bocavirus NP1; IRF9, interferon regulatory factor 9; IFNAR, IFN alpha receptor; TYK2, tyrosine kinase 2; JAK-STAT, Janus kinase–signal transducer and activator of transcription; ISGF3, interferon-stimulated gene factor 3; ISGs, interferon-stimulated genes; ISRE, interferon-stimulated response element.

In the canonical JAK–STAT pathway (Figure 1), binding of type I IFNs to its receptors (IFNAR1 and IFNAR2) leads to the dimerization of both IFNARs [reviewed in Ref. (36)]. This in turn phosphorylates IFNAR1-bound tyrosine kinase 2 (TYK2) which then phosphorylates IFNAR2-bound Janus kinase 1 (JAK1). Then, the receptor-bound kinases phosphorylate STAT1 and STAT2 at amino acid position 701 and 690, respectively. The phosphorylated STAT1 and STAT2 subsequently dimerizes *via* reciprocal SH2-phosphotyrosine interactions [reviewed in Ref. (36)]. Phosphorylated STAT1–STAT2 heterodimer then dissociates from the receptors and recruit IRF9 to form the ISGF3 complex in cytoplasm. ISGF3 will translocate into the nucleus and binds to the promoter region of interferon-stimulated response element (ISRE) to activate the transcription of ISGs (37).

Equally as intriguing, a study has shown that unphosphorylated-ISGF3 (U-ISGF3)—where STAT1 and STAT2 proteins are not phosphorylated—can also induce antiviral effect (38). Nonetheless, a different subset of ISGs was induced by U-ISGF3 compared to those of ISGF3. The U-ISGF3 is suggested to prolong the antiviral response for days beyond the resolution of viral infection (38). The prolonged expression of this subset of ISGs induced by U-ISGF3 ameliorates the response toward IFN-α in HCV-infected liver (39).

## IRF9 Structure

**Figure 2:**
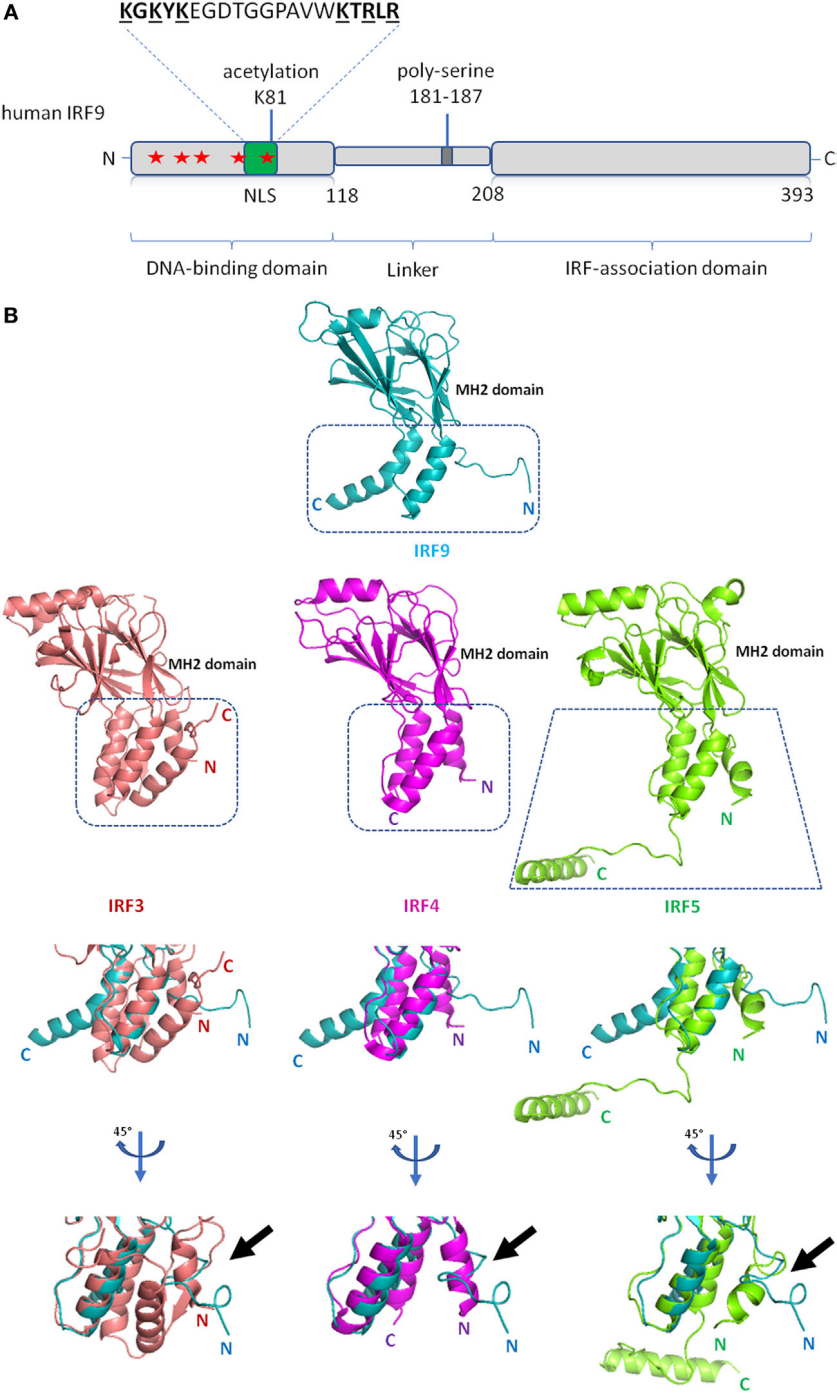
Schematic diagram of interferon regulatory factor 9 (IRF9) and structure of IRF9 IRF-associated domain (IAD). **(A)** Domain organization of the full length human IRF9 shown in a schematic representation. The conserved tryptophan pentad (labeled red stars) of IRF9 are located at amino acid positions 15, 30, 42, 62, and 80 within the DNA-binding domain. Green box indicates the position (a.a. 66–85) of nuclear localization signal (NLS) of IRF9. The largely basic bipartite NLS is characterized as KGKYK separated by a spacer sequence of 10 amino acids followed by KTRLR (basic amino acids are shown underlined). **(B)** Crystal structures of the IAD of IRF9 [Protein Data Bank (PDB) ID code 5OEM], IRF3 (PDB ID code 3A77), IRF4 (PDB ID code 5BVI), IRF5 (PDB ID code 3DSH) show similarity in tertiary structure between all four proteins. The Mad-homology 2 fold (β-sheets, center core) is visibly conserved in the IAD of all four IRFs. Close-up structural superposition between IRF9 against IRF3, IRF4, and IRF5 disclose the absence of N-terminal autoinhibitory helical structure (α1 helix) in the IAD of IRF9 (see black arrow). Therefore, IRF9 could be constitutively active.

As with the other IRFs, IRF9 consists of distinctive DBD and IAD that are joined through a linker (Figure 2A). Instead of forming homodimers, IRF9 forms the ISGF3 complex with STAT1 and STAT2, following induction by type I IFNs. Within the ISGF3 complex, the ISRE consensus sequence 5′-A/GNGAAANNGAAACT-3′ at the promoter region of ISGs is jointly recognized by DBDs of IRF9 and STAT1, while STAT2 DBD interacts with non-consensus sequences (40). The crystal structure of IRF1 bound to DNA revealed a helix-turn-helix DBD attaching to the major groove of the DNA GAAA core sequence, with a slight DNA distortion angled toward IRF1 (41). Likewise, the structure of IRF2 bound to DNA revealed the recognition sequence of AANNGAAA, which similarly show DNA distortion toward IRF2 (42). Subsequent studies on crystal structures of IRF3 (43), IRF4 (44), and IRF7 (45) bound to DNA revealed a similar recognition sequence. As the IRF DBDs are well conserved, there is a significant overlap between the ISGF3 and IRF3/5/7-binding motifs and regulation of various ISGs expression (46, 47). Clearly, DNA-based allostery influences the binding efficiency of these IRFs to specific sequences (46). For example, the -NN- dinucleotide sequence between the GAAA repeats is enriched with -CT- for genes induced by ISGF3, but -TG- for IRF3 homodimers (47).

IRF-associated domain mediates the interaction of IRFs to other factors. Unlike DBD, IAD of all IRFs are not well conserved which subsequently confers specificity to different IRFs. The IRF9 IAD is responsible for binding to the coiled-coil domain of STAT2. The structure of mouse IRF9 IAD generally retains the crescent shape of Mad-homology 2 domain fold, resembling IAD of IRF3 (48). Structure-function analysis shows that IRF3, IRF4, IRF5, and IRF7 have an autoinhibitory domain at their respective C-terminal end, which inherently suppresses the transcriptional activity of the proteins (16, 49–51). For IRF3, IRF5, and IRF7, phosphorylation is necessary to mitigate the autoinhibition. For example, the phosphorylation of IRF5 causes protein conformational changes to unveil previously blocked IAD, allowing IRF5 homodimerization and further binding of CREB-binding protein to IRF5 dimer (16). A similar phosphoactivation mechanism is also predicted for IRF3 (16). On the other hand, IRF4 has a flexible autoinhibitory domain that may abrogate the necessity of phosphorylation in IRF4 activation (51). The linker domain of IRF4 is predicted to be in a compact domain-like conformation, and is involved in the regulation of IRF4 (51). That said, while superposition of the IRF9 IAD to IAD of IRF3, IRF4 and IRF5 reveals general structural homology, the autoinhibitory domain was not identified within the IRF9 IAD (Figure 2B) (48). Therefore, it is plausible that IRF9 is constitutively active, whereas post-translational modifications may induce inactivation instead. For example, the phosphorylation of S123, S173, and T180 at the linker domain of IRF3 disrupts its transactivation activity (52).

## IRF9 Regulation

### Regulation by Post-Translational Modification

Major post-translational modifications that regulate innate immune proteins include phosphorylation, polyubiquitination, SUMOylation, acetylation, methylation, and succinylation [reviewed in Ref. (53)]. All three components of ISGF3 are acetylated by the cytoplasmic CREB-binding protein (54). Acetylation of IRF9 at residue Lys81 is required for DNA binding and is critical in the ISGF3 complex formation during antiviral response signaling (54). However, there has been no follow-up reports ever since. All IRF family members involved in antiviral immunity are known to be regulated by phosphorylation, except for IRF9 (13). The absence of autoinhibitory region from the IRF9 IAD crystal structure reaffirmed previous notions that activation by phosphorylation may not be necessary for IRF9’s association with STAT2 (48). That said, an early paper suggested that IRF9 can be phosphorylated constitutively within the DBD in the absence of IFN stimuli (55). Dephosphorylation of IRF9 *in vitro* by calf intestinal phosphatase abolishes ISRE binding, which suggests a function of IRF9 phosphorylation in DNA association (55). This could represent a yet-to-be characterized mechanism regulating the ISGs expression. To the best of our knowledge, there has been no other report pursuing this interesting find. Therefore, the modulation of IRF9 by post-translational modifications ought to be thoroughly investigated for better understanding of this protein.

### Regulation by MicroRNA

Interferon regulatory factor 9 is also subject to regulation by miRNAs such as miR-93 and miR-302d. The inhibition of *IRF9* mRNA by miR-93 results in the decrease of IRG1-itaconic acid, which in turn enhances angiogenesis, arteriogenesis, and perfusion recovery in ischemic muscles (56). On the other hand, monocytes of systemic lupus erythematosus patients have reduced level of miR-302d expression, resulting in increased IRF9 expression (10). Increased expression of type I IFNs and ISGs are among the hallmarks of lupus disease progression (57), consequently leading to high production of IRF9-mediated IgG autoantibodies (58). Nevertheless, *in vivo* transfection of miR-302d mimic was sufficient to reduce ISGs expression *via* inhibition of IRF9-mediated signaling (10).

## IRF9 Protein Interaction Dictates its Other Functions

### IRF9–STAT2

In addition to JAK–STAT pathway, IRF9 was also shown to constitutively bind to STAT2 in the cytoplasm under non-stimulated condition (59) and that it is necessary for regular nuclear-cytoplasm shuttling [reviewed in Ref. (60, 61)]. The interacting domains were initially predicted (62) and mapped to the STAT2 coiled-coil domain (133–315 a.a.) and IRF9 IAD (182–385 a.a.) (48). IRF9 lacks the nuclear export signal while possessing the classical bipartite nuclear localization signal (NLS) between amino acid residues 66 and 85 within its DBD (59). Conversely, STAT2 lacks the NLS but maintains functionality of its nuclear export signal. As a result, in the absence of STAT2, IRF9 localizes in the nucleus (59). The IRF9–STAT2 dimer localizes to the nucleus by interaction of IRF9 NLS to importin-α/importin-β1 complex (60). However, nuclear localization of ISGF3 is mediated by the interaction of STAT1 NLS to importin-α5/importin β1 complex (60, 63). This switch in importin binding is likely due to change in protein conformation. Indeed, a rendered model of ISGF3 bound to DNA (48) indicates the NLS of IRF9 becoming inaccessible due to its protein conformation, whereas the STAT1 NLS is exposed hence allowing for nuclear transporter binding. Interestingly, IRF9 fused with STAT2 transactivation domain alone can induce antiviral state (64). Other studies have also revealed important regulatory functions of IRF9–STAT2, which includes gene expression of retinoic acid-induced gene G (65), prolonging the ISGF3-like transcriptional activity (66) and drives the IL-6 expression (67)—a proinflammatory cytokine whose elevated serum level is associated with various cancers (68). On a different note, one study reported fewer ISGs being expressed in STAT1- or STAT2-deficient murine glial cells compared to IRF9-deficient cells upon IFN-α stimulation, reflecting the dominant role of STATs in non-canonical IFN signaling (69).

### IRF9–Cyclophilin A (CypA)

Proinflammatory cytokines are a subset of ISGs being regulated by IRF9 (70). CypA is a peptidyl-prolyl isomerase involved in the proper folding of proteins and immune cell activation [reviewed in Ref. (71)]. Interestingly, HCV non-structural 5A protein (NS5A) was found to compete with IRF9 for CypA binding *in vitro*, resulting in increased transcriptional activity of IFN-induced ISRE in HepG2 cell lines (72). HCV infection could lead to inflammation and fibrosis in the liver (73). Therefore, the acute liver inflammation associated with early stage of HCV infection may be an inadvertent effect of NS5A sequestration of CypA that is a repressor of IRF9-regulated inflammation. In addition, IRF9-deficient mice were protected from DSS-induced intestinal inflammation, suggesting yet again that IRF9 is pro-inflammation (9).

### IRF9 and Peroxisome Proliferator-Activated Receptor α (PPARα)–Sirtuin1 (SIRT1) Axis

Recently, researchers have linked IRF9 to the poor outcome of ischemic reperfusion (IR) injuries (70, 74, 75). Compared to wild-type mice, mice overexpressing IRF9 developed a more severe myocardial damage and exhibited inflammation when challenged with IR, while a reduced response was noticed in IRF9-knockout mice (70). Whereas, liver cells overexpressing IRF9 underwent apoptosis more readily compared to IRF9-deficient cells when subjected to IR challenge (75). In the study, the authors found that IRF9 suppresses gene expression of *SIRT1* responsible for the inhibition of pro-apoptotic protein, p53. In addition, the suppression of *SIRT1* by IRF9 contributes to neointima formation (76).

**Figure 3:**
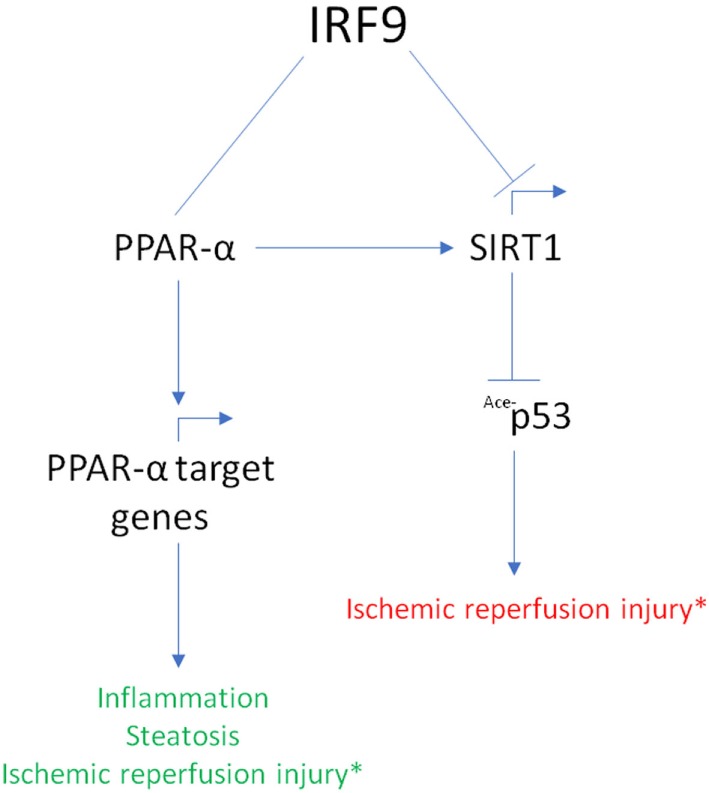
Summary of interferon regulatory factor 9 (IRF9) interaction with the peroxisome proliferator-activated receptor α (PPARα)–Sirtuin1 (SIRT1) axis. IRF9 exerts different effect in its interaction with the PPARα–SIRT1 axis. IRF9 interacts with PPARα and activates PPARα target genes to attenuate inflammation, liver steatosis, and ischemic reperfusion (IR) injury. Whereas, IRF9 inhibits the expression of SIRT1 resulting in augmented acetylation of p53 protein. This results in a poor outcome in IR injury. PPARα is also known to regulate SIRT1 gene expression. *Conflicting roles of IRF9 in the PPARα*–SIRT1 axis result in different outcome in IR injury (green font indicates better outcome; red font indicates worst outcome)*.

Meanwhile, the linker region of IRF9 was shown to interact with PPARα to activate PPARα target genes (77). This interaction was found to reduce steatosis, hepatic IR injury, and inflammation (77). Interestingly, the PPARα–SIRT1 axis has been known to mediate cardiac hypertrophy, metabolic dysregulation, inflammation, and anti-aging pathways (74). Together, these studies uncovered a novel role of IRF9 in IR injury progression, steatosis, and inflammation through interaction with the PPARα–SIRT1 axis (Figure 3). The seemingly conflicting action of IRF9 on PPARα and SIRT1 necessitate further investigation.

### IRF9-Viral Proteins

Massive upregulation of ISGs following activation of the JAK–STAT pathway will establish antiviral state in the infected and neighboring cells. The potency of ISGs against viral infections is highlighted by the many ways viruses have evolved to interfere with IRF9, alone or as part of ISGF3 (Figure 1). IRF9 was specifically antagonized by viruses through nuclear sequestration, inhibiting DNA binding of IRF9 and promoting IRF9 degradation. Human papillomavirus 16 produce E7 oncogenes [reviewed in Ref. (78)] that interacts with IRF9 to prevent ISGF3 complex formation and nuclear translocation (79). This interaction occurs between amino acids 25 and 36 of E7 PEST domain and between 327 and 354 of IRF9 IAD domain (80).

Conversely, reovirus type 1 (strain Lang) (T1L) μ2 protein was found to cause IRF9 nuclear accumulation in the absence of IFN stimulation (81). The authors also hypothesized that the T1L μ2 protein prevents IRF9 binding to STAT2. It is of note that a single change of amino acid 208 of T1L μ2 can repress IFN-β signaling (82). However, detailed mechanism on T1L μ2–IRF9 interaction is yet to be defined.

Varicella zoster virus (VZV) is the causative agent of chickenpox in children and establishes latency in the nervous system to cause herpes zoster (shingles) later in adulthood [reviewed in Ref. (83)]. The ORF63 protein of VZV is present during viral lytic phase and is one of immediate early protein expressed in latently infected human ganglia (84). The simian varicella virus (SVV) infection in rhesus macaques has been used as an animal model of VZV infection (83). A recent study shows the SVV ORF63 protein induces specific degradation of IRF9 in a proteasome-dependent manner (85). In rhesus fibroblast cells expressing ORF63, supplementation with proteasome inhibitor MG132 led to increased cellular level of IRF9 compared to non-treated cells (85).

Porcine bocavirus NP1 protein has been reported to bind to the DBD of IRF9, effectively blocking the binding of ISGF3 complex to ISRE promoter, thus reducing ISGs expression (86).

## Conclusion and Future Directions

Interferon regulatory factor 9 was initially discovered as a component of the potent transcription factor ISGF3 responsible in initiating transcription of hundreds of ISGs to mount antiviral response. IRF9 is further implicated in expansive roles across the pathogenesis and improvement of diseases. Surprisingly, there is limited information on the mechanistic detail of IRF9’s various functions, beyond its association with STAT1 and STAT2. Extensive studies are required to elucidate the regulatory mechanisms that govern the IRF9 transcriptional and translational activities, sequestration by protein binding, and compartmentalization. In particular, the dual function of IRF9 in promoting and reducing inflammation requires further investigation. Although not explicitly discussed here, IRF9 is upregulated by c-Myc protooncogene (4) and cell crowding (87), suggesting involvement of IRF9 in oncogenesis. In addition, general screening of candidate genes revealed that increased expression of IRF9 and XRCC1 as genetic biomarkers are predicative of glioblastoma multiform progression (88).

Similarly, further elucidation of virus–host interactions suppressing IRF9-mediated transcription is also an area of intrigue. The genomic sequence of IRF9, though well conserved among mammals, fish, reptiles, and amphibians, is not found in avians (89). The interplay between other immune-regulatory pathways to compensate for absence of IRF9 in birds may shed additional information about the extensive role of IRF9 in other species. Of note, there is a growing interest in IRF9 studies on its broad impact on the antiviral immunity of fishes (90–95).

The knowledge of IRF9 beyond ISGF3 is still at its nascent stage, thus further studies are necessary to explore the molecular function and implication of this key protein in antiviral immunity and beyond. The recent advances in IRF9’s structural information will provide better insights in future studies focusing on its wide-ranging function and regulatory role.

## Author Contributions

AP and SKN prepared the draft manuscript. AP, THT, and SKN revised and edited the final manuscript.

## Conflict of Interest Statement

The authors declare that the research was conducted in the absence of any commercial or financial relationships that could be construed as a potential conflict of interest.
